# Comparison of early-, late-, and non-participants in a school-based asthma management program for urban high school students

**DOI:** 10.1186/1745-6215-12-141

**Published:** 2011-06-06

**Authors:** Christine LM Joseph, Jacquelyn Saltzgaber, Suzanne L Havstad, Christine C Johnson, Dayna Johnson, Edward L Peterson, Gwen Alexander, Mick P Couper, Dennis R Ownby

**Affiliations:** 1 Department of Biostatistics and Research Epidemiology, Henry Ford Health System, Detroit, MI, USA; 2 Institute for Social Research, University of Michigan, Ann Arbor, MI, USA; 3 Clinical Allergy and Immunology, Medical College of Georgia, Augusta, GA, USA

## Abstract

**Background:**

To assess bias and generalizability of results in randomized controlled trials (RCT), investigators compare participants to non-participants or early- to late-participants. Comparisons can also inform the recruitment approach, especially when working with challenging populations, such as urban adolescents. In this paper, we describe characteristics by participant status of urban teens eligible to participate in a RCT of a school-based, web-based asthma management program.

**Methods:**

The denominator for this analysis was all students found to be eligible to participate in the RCT. Data were analyzed for participants and non-participants of the RCT, as well as for students that enrolled during the initially scheduled recruitment period (early-participants) and persons that delayed enrollment until the following fall when recruitment was re-opened to increase sample size (late-participants). Full Time Equivalents (FTEs) of staff associated with recruitment were estimated.

**Results:**

Of 1668 teens eligible for the RCT, 386 enrolled early, and 36 enrolled late, leaving 1246 non-participants. Participants were younger (p < 0.01), more likely to be diagnosed, use asthma medication, and have moderate-to-severe disease than non-participants, odds ratios (95% Confidence Intervals) = 2.1(1.7-2.8), 1.7(1.3-2.1), 1.4(1.0-1.8), respectively. ORs were elevated for the association of late-participation with Medicaid enrollment, 1.9(0.7-5.1) and extrinsic motivation to enroll, 1.7(0.6-5.0). Late-participation was inversely related to study compliance for teens and caregivers, ORs ranging from 0.1 to 0.3 (all p-values < 0.01). Early- and late-participants required 0.45 FTEs/100 and 3.3 FTEs/100, respectively.

**Conclusions:**

Recruitment messages attracted youth with moderate-to-severe asthma, but extending enrollment was costly, resulting in potentially less motivated, and certainly less compliant, participants. Investigators must balance internal versus external validity in the decision to extend recruitment. Gains in sample size and external validity may be offset by the cost of additional staff time and the threat to internal validity caused by lower participant follow-up.

**Trial Registration:**

ClinicalTrials.gov: NCT00201058

## Background

When conducting randomized trials, it is standard practice to assess external validity by comparing participants to non-participants [1]. Also suggested is a comparison of early- and late-participants; since late-participants would have been non-participants had recruitment not been extended [2,3]. These descriptions can also be used to refine or modify recruitment approaches, especially when recruiting from challenging populations, such as urban adolescents [1].

Based on our experiences and the experiences of others, adolescents and urban minority populations can be difficult to engage in research [4-8]. Although public high schools represent a large pool of potential adolescent participants, navigating issues of access and consent can be challenging [9]. The requirement for written parental consent has been shown to impact external validity in research studies, especially for schools in underserved communities [10-12]. For urban, low income and minority populations, recruitment can also be influenced by issues of mistrust, differential access to resources (e.g., health care, Internet), and a lack of cultural awareness on the part of investigators [8,13].

The goal of this paper is to describe the characteristics of participants and non-participants for a randomized controlled trial (RCT) of a school-based, online asthma management program for urban teenagers (NHLBI Grant#HL068971) and to estimate staff cost of recruitment. Analyses such as these can provide information on how youth make initial decisions to participate in studies, how motivation may impact their continued involvement, and the extent to which study findings may be biased due to under-representation of certain subgroups. This information could inform other investigators conducting research in difficult-to-engage populations [14-16].

## Methods

All study methods were approved by the Henry Ford Health System Institutional Review Board. To identify students eligible for the RCT, caregivers of all 9th-12th graders of six public high schools were informed by mail of a screening questionnaire on respiratory symptoms to be administered during a regularly scheduled English class. Caregivers could refuse to have their student participate in the screening questionnaire by signing and returning the letter to the school. To maintain student confidentiality, mailings informing caregivers of the screening questionnaire were conducted by a public school vendor authorized to handle student data processing. In this way, investigators were not privy to student information prior to obtaining written, informed consent [17]. All completed screening forms were returned directly to the vendor who applied an eligibility algorithm developed by investigators [17]. Students meeting a priori criteria for current asthma (i.e., report of asthma symptoms with or without report of a physician diagnosis of asthma) were eligible to enroll in the RCT with required written parental consent and the student's assent [18]. Invitations to enroll were mailed by the public school vendor. Students could also request an enrollment packet from school staff or call a special number to find out if they were eligible. Because the identity of eligible students was withheld from researchers until full consent/assent was obtained, recruitment activities were aimed at the entire student body. A variety of recruitment activities were employed to promote study awareness and encourage enrollment of those eligible, including mailings, contests, giveaways, and presentations during selected classes. The initial recruitment period began September 2007 and continued through June 2008. Falling short of our recruitment goal, recruitment was re-opened in the fall of 2008.

Using computers at school, participation included an online baseline survey after which students were randomized to the treatment (tailored, web-based asthma education) or control group (existing web-based, generic asthma education). A stratified block randomization method using three strata (school, sex and asthma severity) was employed. Randomization using random block sizes of 2, 4 and 6 were generated using a computer program.

Following baseline and randomization, students were asked to complete four online asthma management sessions a minimum of 1 week apart and follow-up surveys at 6 and 12 months post-baseline. Caregivers completed a baseline telephone interview (to correspond with student's baseline) and a 12-month interview. As the study progressed, staff effort was split between recruitment and study enrollment duties (e.g., collecting consent forms and guiding students through the online baseline survey). Students were offered cash incentives for enrollment, session completion milestones, and for completing follow-up surveys. Participating students were also able to earn out-of-class-learning credits toward graduation.

At program end, students were asked about their motivation to enroll and could select from a menu of choices. Answers were categorized as representing intrinsic motivation, i.e., actions that result from conscious choice and are personally relevant (e.g., "Wanted to learn to control asthma", "Wanted to learn more about asthma" or "Seemed like a fun program"); as opposed to extrinsic motivation, i.e., actions resulting from a desire for external rewards or actions based on encouragement from another person, such as a caregiver, physician, or friend (e.g., "Gift cards or cash" or "Encouraged by others to enroll").

Student's t test and Chi square test were used for continuous variables and categorical variables, respectively, when comparing student characteristics to participant category. Logistic regression was used to calculate Odds Ratios (OR) and corresponding 95% Confidence Intervals to examine the independent association of selected student characteristics to participant category, using non-participant status as the dependent variable [19,20]. Due to small sample size, multivariate analyses were not conducted for comparisons involving late-participants. All analyses were performed using SAS 9.2 TS Level 1M2, Windows Version 5.2.3790. To estimate the staff time used in conducting recruitment activities, staff hours were retrieved for the recruitment period. When multiple tasks were being conducted during that period, staff hours were multiplied by the documented fraction of time spent specifically on recruitment activities. This was then divided by the number of hours in a given timeframe (e.g., 6 months) to obtain Full Time Equivalents (FTEs), using 2080 as the total number of hours in 12 months. Estimates do not include supervisory staff.

## Results

The recruitment goal was 450 students. Across the six schools, 98% of students were African-American and 74% qualified for free/reduced price lunch. Of the 9125 students enrolled in an English class and present on the day of questionnaire administration, a total of 7878 (86.3%) completed the screening form. Of these, 1668 (21.2%) were eligible for the RCT, of which 1095 (71.7%) reported a diagnosis of asthma, while 573 (9.5%) met symptom criteria for eligibility, but did not report a physician diagnosis of asthma. We were able to capture 29.9% of the eligible diagnosed (327/1095) and 16.6% of those eligible with symptoms and no diagnosis (95/573).

During the initial recruitment period (9/2007-6/2008), 386 students enrolled (early-participants). Another 36 students enrolled when recruitment was re-opened in fall of 2008 (late-participants). There were 1246 students who met eligibility criteria based on responses to the screening questionnaire, but did not enroll (non-participants).

Table 1 shows student characteristics by participant status. Table 2 shows risk estimates representing the association of student characteristics with participant category. At screening, the mean age for participants was significantly lower than that of non-participants, p = 0.017 (Table 1). Participants were also more likely to report a diagnosis of asthma, OR = 2.1 (1.7-2.8), p < 0.01, use medication for respiratory symptoms, OR = 1.7 (1.3-2.1), p < 0.01, and have symptom frequencies consistent with moderate-to-severe asthma, OR = 1.4 (1.0-1.8), p = 0.04 (Table 2). To identify variables independently associated with non-participant status, a logistic regression analysis was conducted using sex, age, grade, asthma diagnosis, medication use, asthma severity, ED visits in the last 12 months, smoking status of student, and student exposure to ETS at home. Results showed that young age, OR = 0.86 for every year increase in age (0.79-0.95), p = 0.003; asthma diagnosis, OR = 2.0 (1.5-2.6), p < 0.001; and medication use, OR = 1.4 (1.1-1.8), p = 0.007 were significant predictors of participation. Moderate-to-severe asthma was of borderline significance OR = 1.3 (1.0-1.7), p = 0.09 (data not shown).

**Table 1:** Characteristics of high school students eligible to enroll in a randomized trial, by participant status

	**PARTICIPANTS**									**NON-PARTICIPANTS**		
	**All (n = 422)**			**Early (n = 386)**			**Late (n = 36)**			**(n = 1246)**		
	
**SCREENING ^1^**	**n**	**Mean**	**(sd)**	**n**	**Mean**	**(sd)**	**n**	**Mean**	**(sd)**	**n**	**Mean**	**(sd)**
	
Mean age (s.d.)	422	15.9	1.1	386	15.9	1.1	36	15.6	1.1	1201	16.1	1.5
	**N**	**n**	**(%)**	**N**	**n**	**(%)**	**N**	**n**	**(%)**	**N**	**n**	**(%)**
Male	422	170	(40.2)	386	152	(39.4)	36	18	(50.0)	1202	487	(40.5)
Senior in High School ^2^	422	55	(13.0)	386	53	(13.7)	36	2	(5.6)	1201	194	(16.1)
Diagnosed asthma	422	327	(77.5)	386	301	(78.0)	36	26	(72.2)	1246	768	(61.6)
Medication used	404	269	(66.6)	370	246	(66.5)	34	23	(67.7)	1162	630	(54.2)
Mod/Severe Asthma	415	88	(21.2)	380	81	(21.3)	35	7	(20.0)	1210	201	(16.6)
≥1 ED visit/12 months	401	190	(47.4)	368	172	(46.7)	33	18	(54.5)	1181	506	(42.9)
Smoker ^3^	416	30	(7.2)	381	30	(7.9)	35	0	(0)	1191	75	(6.3)
Household ETS ^4^	412	263	(63.8)	378	239	(63.2)	34	24	(70.6)	1187	723	(60.9)
**BASELINE **^5^												
Medicaid enrollee	405	297	(73.3)	375	272	(72.5)	30	25	(83.3)			
Extrinsically motivated ^6^	289	40	(9.5)	265	35	(13.2)	24	5	(20.8)			
Rescue inhaler nearby ^7^	191	86	(45.0)	176	80	(45.4)	15	6	(40.0)			
Controller med adherence ^8^	143	39	(27.3)	132	32	(27.3)	11	3	(27.3)			
**STUDY COMPLIANCE **												
**Student**												
All 4 sessions completed	422	373	(88.4)	386	347	(89.9)	36	26	(72.2)			
12 month follow-up completed	422	344	(81.5)	386	328	(85.0)	36	16	(44.4)			
**Caregiver**												
Baseline phone survey completed	422	358	(84.8)	386	338	(87.6)	36	20	(55.6)			
12 month phone survey completed	422	328	(77.7)	386	312	(80.8)	36	16	(44.4)			

^1^Screening questionnaire administered 2007-2008; ^2 ^Senior in high school when Lung Health Survey conducted (2007-2008); ^3 ^Smoked 1 or more days in past 30 days; ^4 ^Environmental tobacco smoke; ^5 ^Information from enrollment baseline survey; ^6 ^Reason for enrollment: Intrinsically motivated ="Wanted to learn to control asthma" or "Seemed like a fun program" vs. Extrinsically motivated = "Gift cards or cash" or "Encouraged by others to enroll". ^7^Among those reporting a rescue inhaler, report having rescue inhaler nearby >4 of last 7 days; ^8 ^Among those reporting a controller medication, report taking controller medication >4 days of last 7 days.

**Table 2:** Comparison of characteristics of teenagers eligible for a randomized trial intervention, by participant status

	**Non-participants vs. All **			**Non-participants vs. Early**			**Non-participants vs. Late**			**Late vs. Early**		
	
**SCREENING ^1^**			***p***			***p***			***p***			***p***
	
Mean age			<0.01			<0.01			0.02			0.18
	
	**OR**	**(95%CI)**	***p***	**OR**	**(95%CI)**	***p***	**OR**	**(95%CI)**	***p***	**OR**	**(95%CI)**	***p***
	
												
Male	1.0	(0.8-1.2)	0.93	1.0	(0.8-1.2)	0.69	1.5	(0.8-2.9)	0.26	1.5	(0.8-3.1)	0.18
Senior in High School ^2^	0.8	(0.6-1.1)	0.13	0.8	(0.6-1.1)	0.25	0.3	(0.1-1.3)	0.11	0.4	(0.1-1.6)	0.43
Diagnosed asthma	2.1	(1.7-2.8)	<0.01	2.2	(1.7-2.9)	<0.01	1.6	(0.8-3.4)	0.20	0.7	(0.3-1.6)	0.89
Medication used	1.7	(1.3-2.1)	<0.01	1.7	(1.3-2.1)	<0.01	1.8	(0.9-3.7)	0.13	1.1	(0.5-2.2)	0.86
Mod/Severe Asthma	1.4	(1.0-1.8)	0.04	1.4	(1.0-1.8)	0.04	1.3	(0.5-2.9)	0.60	0.9	(0.4-2.2)	0.39
≥1 ED visit/12 months	1.2	(0.9-1.5)	0.11	1.2	(0.9-1.5)	0.19	1.6	(0.8-3.2)	0.18	1.4	(0.7-2.8)	0.98
Smoker ^3^	1.2	(0.7-1.8)	0.52	1.0	(0.5-2.0)	0.89	Not calculated (zero cell)			Not calculated (zero cell)		
Household ETS ^4^	1.0	(0.5-1.9)	0.89	1.1	(0.9-1.4)	0.42	1.5	(0.7-3.3)	0.26	1.4	(0.6-3.0)	0.39
												
**BASELINE^5 ^**												
Medicaid enrollee										1.9	(0.7-5.1)	0.20
Extrinsically motivated ^6^										1.7	(0.6-5.0)	0.31
Rescue inhaler nearby ^7^										0.8	(0.3-2.3)	0.68
Controller med adherence ^8^										1.0	(0.3-4.0)	0.99
**STUDY COMPLIANCE**												
**Student**												
All 4 sessions completed										0.3	(0.1-0.7)	<0.01
12 month follow-up completed										0.1	(0.04-0.2)	<0.01
**Caregiver**												
Baseline survey completed										0.2	(0.1-0.3)	<0.01
12 month survey completed										0.2	(0.1-0.4)	<0.01

^1^Screening questionnaire administered 2007-2008; ^2 ^Senior in high school when Lung Health Survey conducted (2007-2008); ^3 ^Smoked 1 or more days in past 30 days;

^4 ^Environmental tobacco smoke; ^5 ^Information from enrollment baseline survey; ^6 ^Reason for enrollment: Intrinsically motivated ="Wanted to learn to control asthma" or "Seemed like a fun program" vs. Extrinsically motivated = "Gift cards or cash" or "Encouraged by others to enroll". ^7^Among those reporting a rescue inhaler, report having rescue inhaler nearby >4 of last 7 days; ^8 ^Among those reporting a controller medication, report taking controller medication >4 days of last 7 days.

In other comparisons, late-participants were also younger than non-participants, mean age = 15.6 (±1.1) and 16.1 (±1.5) years for late versus non-participants, respectively, p = 0.02. Fewer late-participants (5.6%) were high school seniors compared to non-participants (16.1%), OR = 0.3 (0.1-1.3), p = 0.11, although this comparison was not significant (Table 1). Approximately 11% of young males (< 17 years) were late-participants, followed by older males (9.3%), young females (8.4%) and older females (4.7%) (data not shown). None of the late-participants reported smoking.

Using baseline and follow-up data to compare late- to early-participants, elevated ORs were observed for Medicaid enrollment, OR = 1.9 (0.7-5.1), p = 0.20, and extrinsic motivation to enroll in the RCT, OR = 1.7 (0.6-5.0), p = 0.31 (Table 2). Baseline behaviors targeted in the intervention (having a rescue inhaler nearby and controller medication adherence) did not differ by whether or not the student was a late- versus early-participant, OR = 0.8 (0.3-2.3), p = 0.68, and 1.0 (0.3-4.0), p = 0.99, respectively (Table 2). As shown in Table 2 compared to early participants, late-participants had significantly poorer compliance for completion of the computer sessions, OR = 0.3 (0.1-0.7), p < 0.01 and 12 month follow-up, OR = 0.1 (0.04-0.2), p < 0.01. Compared to caregivers of early participants, caregivers of late-participants also were less compliant with completion of the baseline survey, OR = 0.2 (0.1-0.3), p < 0.01 and the 12 month follow-up, OR = 0.2 (0.1-0.4), p < 0.01.

Overall estimated staff time in FTEs for recruitment activities was 1.95 FTEs or a total of 1798 recruitment hours. About 1.74 FTEs was used for the planned enrollment period (early-participants) and another 0.22 (or 230 hours) was used for extended enrollment. As a representation of retention costs resulting from prolonged recruitment, we calculated the staff hours and expenditures for the period between the date of the last follow-up completed by early-participants (12/14/09) and the last follow-up completed by late-participants (3/21/10). One early-participant who completed a 12 month follow-up on 3/26/10 was included in these analyses. During this period, we estimated 673.5 hours were spent on retention and follow-up of late-participants or another 0.97 FTEs. Total FTEs for extended enrollment was 0.22+0.97 = 1.19, approximately 41% of total recruitment FTEs. We calculate that 0.45 FTEs were needed per 100 recruited for early-participants and 3.3 FTEs were needed per 100 recruited for late-participants.

## Discussion

A comparison of participants to non-participants is done to assess bias and generalizability of study findings, but may also inform future recruitment strategies [1,3]. Based on this analysis, we found that learning more about a personal health condition was a better motivator than cash incentives, and that extending recruitment, while increasing sample size, resulted in enrollment of less motivated students who were less compliant with the study protocol. Future studies might focus on different types of incentives and on finding ways to tailor (individualize) recruitment messaging.

Although non-participants were older in our study, compared to early-participants, a higher percentage of late-participants were young males, perhaps representing a subgroup in denial or reluctant to reveal that they have asthma [21]. According to the literature, adolescent unease about being labeled as having asthma is less a concern for females then for males, and adolescent males with asthma have a higher tendency toward denial than adolescent females [21,22]. A different recruitment approach may work best for this group. For example, recruitment of African-American men improved in a prostate cancer study when lay recruiters of like race conducted 1-on-1 interviews with potential participants [23].

Late-participants and their caregivers were less compliant than other groups [24]. Economic difficulties may interfere with follow-up as suggested by a greater number of Medicaid recipients in this group [24]. Extrinsic motivation, more characteristic of late-responders, is one aspect of an "external locus of control" which was associated with attrition in a recent study of adults [25].

To our knowledge, few US studies of early versus late enrollment among urban adolescents are available for comparison. One US school-based study, the High 5 Alabama study, did compare participants to non-participants and reported that certain subpopulations were under-represented in the study sample [4]. Mattila et al, found significant differences between survey respondents and nonrespondents when following a cohort of Finnish adolescents aged 12-18 years. Nonrespondent males had increased risk of violence-related and unintentional injuries compared to respondents [26]. Vinther-Larsen et al reported that male sex and factors related to low socioeconomic status were related to survey response, as were reports of drinking and smoking, in a Danish cohort of over 12,000 adolescents recruited in grade 7 [27]. Hille et al assessed nonresponse to telephone contact and survey nonresponse bias in a follow-up survey of 19 year old adolescents born as pre-term infants, and found that males were less likely to respond, as were adolescents whose mothers had low education [28]. Investigators emphasized the need for more studies characterizing study non-participants and survey/phone nonresponders in adolescent populations, and describing the impact of nonparticipantion/response on the generalizability of research findings in this age group.

A typical limitation of studies on nonresponse is the lack of information on non-participants and this was true of our study [3]. Also, a small number of late-participants prevented multivariable analyses in this group. Therefore, some of our speculations above are based on measures of effect and not statistical significance. In greater numbers, late-participants may have changed the overall profile of the study population, although this has not been reported by other empirical studies with larger numbers of late-responders [2,3,29].

Falling short of our recruitment goal by 6% (enrolling 422 of the targeted 450), recruitment of urban teens with asthma to a RCT proved challenging. Extending enrollment increased our sample by 8.5%, although the FTEs required for extending recruitment were 41% of the total FTEs used for the study. Our increase in sample size must also be weighed against the cost of additional follow-up efforts due to poor compliance among late-participants [3]. Moreover, the potential for bias is increased if late-responders are very different from non- and early-responders, as suggested in one simulated study specifically evaluating prolonged recruitment [30].

## Conclusions

According to our findings, students meeting symptom frequency criteria consistent with moderate-to-severe asthma were significantly more likely to participate. Because symptom frequency often reflects asthma that is under-managed [31], we believe that recruitment messages were successful in attracting those students struggling with asthma control. In addition, a higher percentage of early participants were intrinsically-motivated. This information can be used in conjunction with the analysis of RCT outcomes (e.g., reductions in asthma morbidity). According to the Self-Determination Theory, intrinsic motivation is highly predictive of positive behavior change [32]. If the study population comprises participants inherently moved to change, overall results of the trial will be favorable. Knowing something about the characteristics of the non-participants allows a more informative application of this theory as well. Finally, "tailored recruitment", or the ability to adapt or alter recruitment messages to an individual's values, goals, or interests, may be more effective in reaching subpopulations difficult to engage.

For health surveys, it has been suggested that the most cost-effective and unbiased approach to recruitment is to begin with a large pool of eligible persons, and accept a lower response rate and reduced generalizability in return for a better follow-up rate [2,3]. Our analysis suggests this may be the best possible approach for behavioral interventions as well.

## List of abbreviations

RCT: Randomized controlled trials; FTE: Full Time Equivalents; CI: Confidence Interval; OR: Odds Ratio.

## Competing interests

The authors declare that they have no competing interests.

## Authors' contributions

CLMJ was Principal Investigator of the study and wrote the manuscript; JS was project coordinator for the study, collected, managed, and retrieved data for the manuscript, and was involved in drafting and reviewing the final manuscript; SH conducted all analyses for the manuscript and reviewed the manuscript for statistical and intellectual content; CCJ was a co-Investigator on the study and reviewed the manuscript critically for important intellectual content; DJ assisted in coordination of the study, in the retrieval and preparation of data for the manuscript, and in critical review of the final manuscript; EP was a co-Investigator on the study and reviewed the manuscript for statistical and intellectual content; GA was a co-Investigator on the project and reviewed the manuscript for intellectual content; MC was a consultant on the project, provided guidance on manuscript preparation, and critically reviewed the manuscript for intellectual content; DO was a co-Investigator on the project and reviewed the manuscript for intellectual content. All authors read and approved the final manuscript.
